# Ethnoecological, Elemental, and Phytochemical Evaluation of Five Plant Species of Lamiaceae in Peshawar, Pakistan

**DOI:** 10.1155/2020/2982934

**Published:** 2020-08-25

**Authors:** Sulaiman, S. M. Shah, Sadaf, M. Amin, B. Gul, M. Begum

**Affiliations:** ^1^Department of Botany, University of Peshawar (UoP), Peshawar, KP 25120, Pakistan; ^2^Centre of Plant Biodiversity, University of Peshawar (UoP), Peshawar, KP 25120, Pakistan

## Abstract

The use of medicinal plants as an unconventional health treatment is gaining considerable recognition and popularity worldwide. The current study was designed to inspect five medicinally important species (such as *Mentha longifolia* (L.) Huds.*, Mentha piperita* L.*, Mentha spicata* L.*, Ocimum basilicum* L., and *Rosmarinus officinalis* L.) of Lamiaceae, collected from district Peshawar, through ethnoecological, phytochemical, and elemental analyses. Biological spectra expressed that therophytes (60%) were the dominant life-form class, while nanophyll (60%) was the leading class among leaf size. The ethnobotanical profile showed that all the species were medicinal and ornamental (100%) each, while 60% were used in spices. Quantitative analysis for the macro- and microminerals confirmed the presence of 13 elements (C, N, O, Mg, K, P, S, Ca, Al, Si, Fe, Cl, and Na), which were present in varying amounts from species to species. The methanol extract of leaf samples was used for the analysis of phytochemical constituents such as saponins, flavonoids, tannins, terpenoids, phlobatannins, steroids, and anthraquinones. The medicinal potential of these plants was correlated with the presence of these phytochemicals. Due to the presence of active constituents, the plants had high potential in antifungal, antidiuretic, antioxidant, and anti-inflammatory activities.

## 1. Introduction

**Figure 1:**
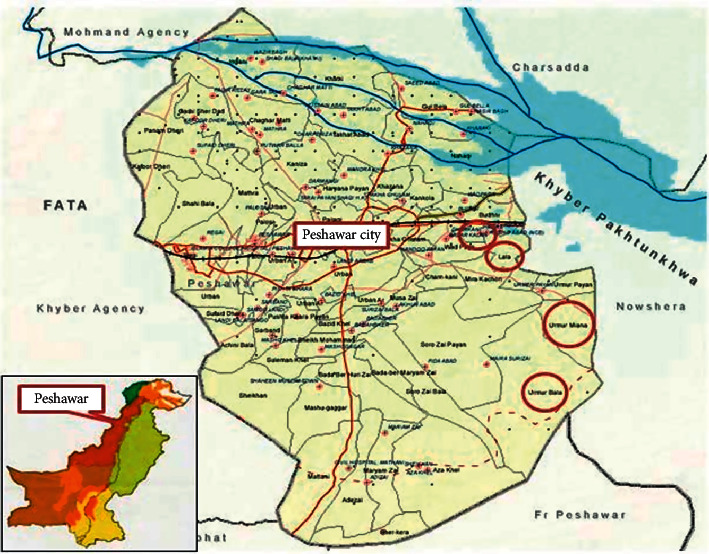
Map of the study area. Source: Geography Department, University of Peshawar, KP, Pakistan.

Peshawar is the capital city of Khyber Pakhtunkhwa, province of Pakistan, located at the altitude of 300 meters between 35°50′37N latitude and 71°21′45E longitude (Figure 1). Its population is 1,970,042, and it covered an area of 1,257 kilometer square [1]. Edaphology deals with the influence of soil on living things specially plants. Peshawar is covered with the combined deposits of sand, silt, and loamy soil, which support a great diversity of flora. Peshawar is not located in the monsoon region unlike the other northern parts of Pakistan. The area has extreme conditions; the coldest month is January with average maximum temperature 18.35°C, and the hottest month is June having a mean maximum temperature of 40.8°C. The relative humidity varies from 46% in June to 76% in August [2]. The average annual rainfall recorded was 454.2 mm. Plants are used as medicine and food since time immemorial round the globe due to their most valuable properties. Medicinal plants play an important role in drug discovery, and human beings used them for various purposes from ancient time.

About 80% of the population in the developing countries depends on medicinal plants for primary health care [3]. Out of 50,000 angiospermic plants used as medicines, more than 600 species are used in Pakistan for curing various diseases [4, 5]. The family Lamiaceae, also known as Labiatae or mint family, consists of highly valued medicinal plants with cosmopolitan distribution and worldwide source of spices and various extracts [6]. Within this family, about 236 genera and more than 6000 species are present in which genera *Ocimum* and *Mentha* provide various taxa [6, 7]. Ethnoecological studies have been carried out by various research studies in Peshawar and adjoining areas [8–14]. The plants of this family contain active chemical constituents and secondary metabolites like vitamins and minerals [15]. *Rosmarinus officinalis* is used as an antimicrobial, anti-inflammatory, antidiuretic, antidiabetic, and anticancer agent [16]. *Mentha* species possess antioxidant properties because of the presence of active components, such as rosmarinic acid, menthol, carvone, menthone, and flavonoids [17]. Minerals play a primary role in reproduction, growth, health, and proper functioning of living organisms [18]. Elemental analysis showed that members of Lamiaceae taxa are rich in macro- and microelements. Phytochemicals are naturally occurring biologically active compounds found in plants, which are liable for health benefits of humans [19, 20]. The phytochemical screening of various plants of Lamiaceae was carried out by Asghari et al., Cocan et al., and Mahendra and Kakde [21–23] and elemental analysis by Arika et al. and Gogoasa et al. [24, 25].

The aim of the present work was to evaluate the macro- and microelements quantitatively and phytochemical constituents qualitatively. The ecological, ethnobotanical, and ethnomedicinal properties were also known. The findings will help in future research studies.

## 2. Materials and Methods

### 2.1. Collection of Plants

Fresh leaves of five medicinal plants, *M. longifolia, M. piperita, M. spicata, O. basilicum*, and *R. officinalis*, were collected from district Peshawar. Plant specimens were taxonomically identified in the Centre of Plant Biodiversity by Dr. Syed Mukaram Shah (PhD), and voucher specimens were deposited in the Herbarium of Department of Botany, University of Peshawar.

### 2.2. Ecological and Ethnobotanical Profiles

Ecological characteristics of plants depend upon altitude, climate, and related environmental conditions. Life form, leaf size, leaf shape, and phenology of the plants were observed [26–28]. Ethnobotany is the cultural relationship among plants, people, and environment. Ethnobotanical information was gathered through the literature and from the local inhabitants. Ethnomedicinal information was documented from local hakims and aged people.

### 2.3. Phytochemical Analysis

Leaves were shade-dried for three weeks, grinded into powder using an electrical grinder, and kept in polythene bags with proper labeling for further use. 50 grams of each powder sample were added separately into 250 ml of methanol solvent in a conical flask at room temperature. After 48 hours, the extracts were filtered with Whatman No. 1 filter paper. The extracts were stored in air-tight glass at 4°C for further analysis.

In test tubes, 0.5 gram of each plant sample was shaken with 5 ml of distilled water. Frothing which persists on warming was taken as preliminary evidence for the presence of saponins [29].

For flavonoid detection, 5 ml of solution of dilute ammonia was mixed with 0.5 gram of the plant sample followed by the addition of concentrated sulphuric acid. Yellow coloration indicates the presence of flavonoids which disappear later on standing [30].

For the detection of terpenoids, 3 ml of concentrated sulphuric acid and 2 ml of chloroform were added to 0.5 gram of the plant sample to form a layer. Reddish-brown coloration showed the presence of terpenoids [31].

For steroid detection, 10 ml of chloroform was mixed with 1 ml of the plant extract, and equal volume of concentrated H_2_SO_4_ was added by the side of the test tube. The upper film turns red, and the H_2_SO_4_ layer shows yellow with green florescence. This indicates the presence of steroids [32].

0.2 gram of each plant powder was boiled in one percent aqueous HCl solution. The formation of red precipitate indicates the presence of phlobatannins [30, 31].

For the detection of tannins, 0.5 gram of each sample was stirred with 100 ml of distilled water, filtered, and 0.1 percent of ferric chloride reagent was added to the 20 ml filtrate. The appearance of blue-green coloration shows the presence of tannins [33].

For anthraquinone detection, 0.5 gram of the plant extract was shaken with 5 ml of chloroform. The solution was filtered, and 10 percent ammonia solution was added to the filtrate. The mixture was shaken thoroughly, and the formation of pink/violet color in the ammonical phase indicates the presence of anthraquinones [31].

### 2.4. Elemental Analysis

**Figure 2:**
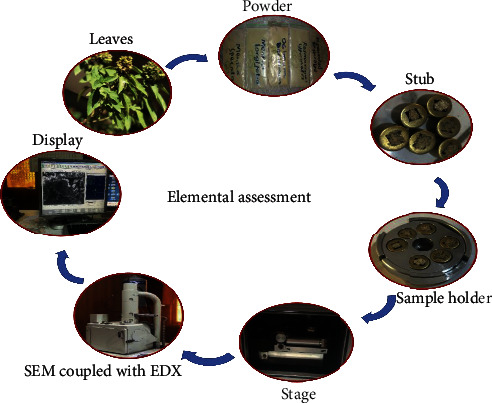
Elemental analysis.

Plants were collected, shade-dried, and ground to powder. For the quantitative analysis of macro- and microelements, energy-dispersive X-ray spectrometer (Model Perkin Elmer AA Analyst 700) was used which is installed in the Centralized Resource Laboratory, University of Peshawar. 0.5 gram of each plant powder was placed on stub, which was grip in the sample holder. The sample holder was laid inside the stage, and the elements' peaks were observed through display (Figure 2). Quantitative results obtained were copied to an Excel worksheet. The worksheet was referred to as raw data. All the data in the raw data worksheet were copied to an edited worksheet where unnecessary data columns were deleted. Also, unnecessary element row lines were removed, leaving the calculated averages as the final concentration data.

## 3. Results and Discussion

### 3.1. Ecological and Ethnobotanical Profiles

**Figure 3:**
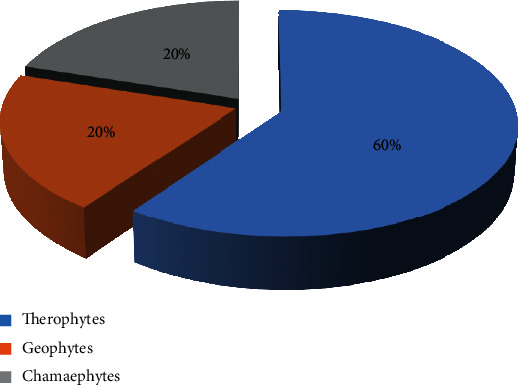
Life-form classes.

During the present research work, 5 plant species of Lamiaceae were collected from the study area which were investigated for their various biological aspects such as phenology, life form, leaf shape, leaf size, and ethnobotanical uses along with phytochemical and elemental screening. During collection time, the phenological stage of *Mentha* species was pre-reproductive, while *Ocimum* and *Rosmarinus* were at reproductive and postreproductive stages (Table 1). Sixty percent species were therophytes, and 20% were each chamaephytes and geophytes (Figure 3). The biological leaf spectra showed that 60% species had nanophyllous and 40% had microphyllous leaves (Table 2). All the species had a simple leaf shape. Ethnobotanical profile showed that a majority of plant species were used in spices and had medicinal and ornamental importance (Table 3). Various parts of the plants contained active constituents such as menthol, menthone, and rosmarinic acids which are used as antifungal, antioxidant, anti-inflammatory, and antidiuretic agents (Table 4). The present work agrees with many other research studies [17, 34], where both reported that these species were used as antioxidant, antidiuretic, and antimicrobial agents.

### 3.2. Macro- and Microelements' Assessment

**Figure 4:**
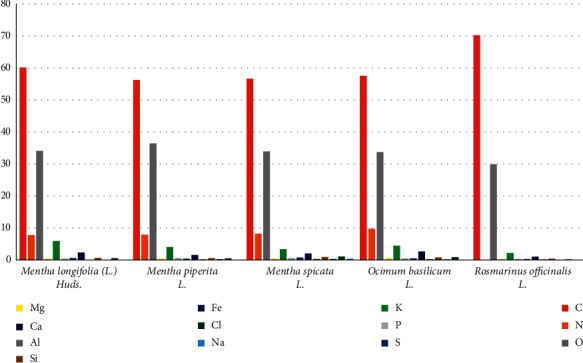
Elemental assessment of plant species: (a) *Mentha spicata* L.; (b) *Mentha longifolia* (L.) Huds.; (c) *Rosmarinus officinalis* L.; (d) *Mentha piperita* L.; (e) *Ocimum basilicum* L.

**Figure 5:**
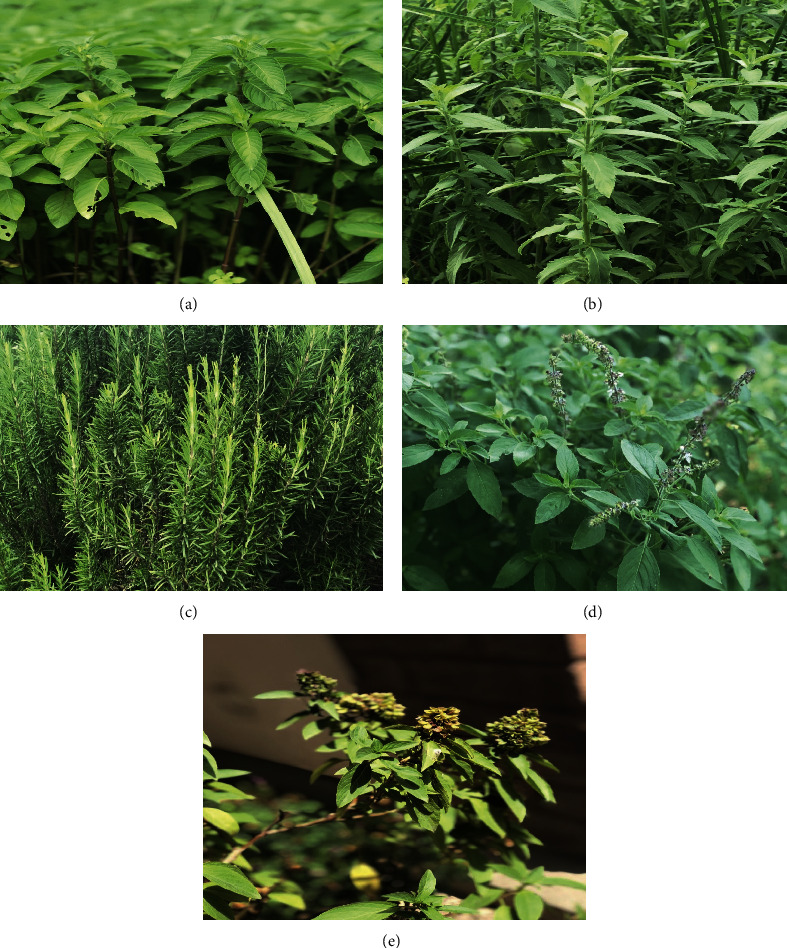
Mentha spp.

All the plant species had high mineral contents and can be used as a good source for medicines (Table 5). A total of 13 different elements were quantitatively analyzed in all the five plant species. Among the major elements, carbon was maximum (70.25%) in *R. officinalis* and minimum (56.2%) in *M. piperita*, while only nitrogen was absent in *R. officinalis*. In minor elements, chlorine was found highest (1.09%) in *Mentha spicata* and lowest (0.19%) in *R. officinalis* (Figures 4 and 5)*. O. basilicum* and *M. longifolia* had high calcium content which plays a key role in bone formation [35]. *M. longifolia* had the highest potassium concentration which plays a key part of many enzymes' synthesis and plays a vital role in the activation of growth of enzymes [36, 37]. Arika et al. [24] and Gogoasa et al. [25] had reported C, N, O, Mg, K, P, S, Ca, Al, Si, Fe, Cl, and Na elements in *Mentha* species, *Ocimum* species, and in *Rosmarinus* species.

### 3.3. Phytochemical Screening

The phytochemical tests of plant extracts were determined for the presence of flavonoids, saponins, phlobatannins, tannins, steroids, terpenoids, and anthraquinones (Table 6). The results showed that the majority of the plants contained flavonoids, terpenoids, and saponins, while anthraquinones were only present in *O. basilicum.* The extract of *M. piperita* and *R. officinalis* contained flavonoids which are used in various ailments including dyspepsia, renal pain, arthritis, and antitumor [38]. Steroids were present in most plant extracts which are of great importance in pharmacy because they possess compounds like sex hormones [39]. The finding agrees with that of Adham [6] and Inas et al. [32], who also observed flavonoids, phlobatannins, saponins, terpenoids, tannins, steroids, and anthraquinones in these species.

## 4. Conclusions

The use of medicinal plants is a traditional practice in Pakistan; thus, it is very important to evaluate the therapeutic use of plants through scientific methods and provide information about the species that could be used in the future for their properties. The medicinal plants appear to be rich in secondary metabolites and mineral contents, widely used in traditional medicine to combat and cure various diseases. The selected five species in this study consist of many useful phytochemical compounds and active elements having important biological properties. Phytochemical investigation and elemental analysis of *M. longifolia, M. piperita, M. spicata, O. basilicum*, and *R. officinalis* showed significant results and may be used for curing of different ailments and manufacturing of new drugs in the future.

## Figures and Tables

**Table 1:** Biological spectra of plants of Lamiaceae in Peshawar, Pakistan.

S. no.	Plant species	Voucher no.	Life form	Leaf size	Leaf shape	Phenology
(A)	Angiosperm					
(a)	Dicots					
(1)	Family Lamiaceae					
(1)	*Mentha longifolia* (L.) Huds.	B.Sul.015.UOP	G	Mic	S	S1
(2)	*Mentha piperita* L.	B.Sul.016.UOP	Th	N	S	S1
(3)	*Mentha spicata* L.	B.Sul.017.UOP	Th	N	S	S1
(4)	*Ocimum basilicum* L.	B.Sul.018.UOP	Th	Mic	S	S2
(5)	*Rosmarinus officinalis* L.	B.Sul.019.UOP	Ch	N	S	S3

Ch = chamaephytes, Th = therophytes, G = geophytes, Mic = microphyll, N = nanophyll, Mes = mesophyll, S = simple, S1 = pre-reproductive, S2 = reproductive, and S3 = postreproductive.

**Table 2:** Summary of biological spectra of plant species of Lamiaceae in Peshawar, Pakistan.

S. no.	Life-form classes	No. of species	Percentage (%)
*(A) Life form*			
(i)	Therophytes (Th)	3	60
(ii)	Chamaephytes (Ch)	1	20
(iii)	Geophyte (G)	1	20
Total		**5**	**100**

*(B) Leaf sizes*			
(i)	Nanophyll	3	60
(ii)	Microphyll	2	40
Total		**5**	**100**

*(C) Leaf shape*			
(i)	Simple	5	100
Total		**5**	**100**

*(D) Phenology*			
(i)	Pre-reproductive (S1)	3	60
(ii)	Reproductive (S2)	1	20
(iii)	Postreproductive (S3)	1	20
Total		**5**	**100**

**Table 3:** Ethnobotanical profile of plant species of Lamiaceae in Peshawar, Pakistan.

S. no.	Plant species	(1)	(2)	(3)
(1)	*Mentha longifolia* (L.) Huds.	+	−	+
(2)	*Mentha piperita* L.	+	−	+
(3)	*Mentha spicata* L.	+	+	+
(4)	*Ocimum basilicum* L.	+	+	−
(5)	*Rosmarinus officinalis* L.	+	+	−
Total		**5**	**3**	**3**
Percentage (%)		**100**	**60**	**60**

(1) = medicinal, (2) = ornamental, and (3) = spices.

**Table 4:** Ethnomedicinal uses of plants of Lamiaceae in Peshawar, Pakistan.

S. no.	Plant species	Part used				Constituents	Diseases
		Root	Stem	Leaves	Flower		
(1)	*Mentha longifolia* (L.) Huds.	**+**	**+**	**+**	**+**	Pulegone, menthone, borneol, and flavonoids.	Carminative stimulant, antispasmodic, and headache.
(2)	*Mentha piperita* L.	−	−	**+**	**+**	Menthol, menthone, methyl acetate, and steroids.	Irritable bowel syndrome, muscle pain, nerve pain, and itching.
(3)	*Mentha spicata* L.	−	−	**+**	−	Terpenoid, carvone, menthol, menthone, and tannins.	Antitumor, antioxidant, and antimicrobial.
(4)	*Ocimum basilicum* L.	**+**	−	**+**	**+**	Saponins, coumarins, anthocyanin, and terpenoids.	Antioxidant, antifungal, and antiviral.
(5)	*Rosmarinus officinalis* L.	−	−	**+**	−	Rosmarinic acid, camphor, flavonoids, and saponins.	Antioxidant, antidiuretic, and anti-inflammatory.

**Table 5:** Mineral contents in plant species of Lamiaceae in Peshawar, Pakistan.

S. no.	Plant species	Minerals (%)												
		Major elements					Minor elements							
		C	N	O	Mg	K	P	S	Ca	Al	Si	Fe	Cl	Na
(1)	*Mentha longifolia* (L.) Huds.	60.19	7.74	34.07	0.37	5.90	0.40	0.61	2.30	—	0.61	—	0.54	—
(2)	*Mentha piperita* L.	56.26	7.89	36.42	0.38	4.01	0.59	0.40	1.53	0.17	0.54	0.24	0.50	—
(3)	*Mentha spicata* L.	56.69	8.23	33.90	0.39	3.41	0.59	0.76	2.01	0.31	0.93	0.29	1.09	0.39
(4)	*Ocimum basilicum* L.	57.59	9.78	33.72	0.58	4.46	0.46	0.52	2.67	0.25	0.75	0.17	0.89	—
(5)	*Rosmarinus officinalis* L.	70.25	—	29.91	0.27	2.15	0.27	0.28	1.05	0.14	0.40	—	0.19	—

**Table 6:** Phytochemical screening of plants of Lamiaceae in Peshawar, Pakistan.

S. no.	Plants species	Flavonoids	Steroids	Tannins	Terpenoids	Saponins	Phlobatannins	Anthraquinones
(1)	*Mentha longifolia* (L.) Huds.	+	−	−	+	+	−	−
(2)	*Mentha piperita* L.	+	+	+	−	+	−	−
(3)	*Mentha spicata* L.	−	+	+	+	−	+	−
(4)	*Ocimum basilicum* L.	+	+	−	+	+	−	+
(5)	*Rosmarinus officinalis* L.	+	−	+	+	+	−	−

## Data Availability

The data used to support the findings of this study are included within the article.
